# Long-term HbA1c variability and the development and progression of diabetic retinopathy in subjects with type 2 diabetes

**DOI:** 10.1038/s41598-021-84150-8

**Published:** 2021-02-26

**Authors:** Han Ul Kim, Sung Pyo Park, Yong-Kyu Kim

**Affiliations:** Department of Ophthalmology, Kangdong Sacred Heart Hospital, Hallym University College of Medicine, 150, Seongan-ro, Gangdong-gu, Seoul, 05355 South Korea

**Keywords:** Eye diseases, Endocrine system and metabolic diseases

## Abstract

This study aimed to investigate whether long-term HbA1c variability is associated with the development and progression of diabetic retinopathy (DR) in subjects with type 2 diabetes. We retrospectively reviewed 434 type 2 diabetes subjects without DR who underwent regular DR screening. We reviewed fundus findings, collected HbA1c levels, and calculated the coefficient of variation (CV) and average real variability (ARV) of each subject’s HbA1c level. DR was developed in 55 subjects and progressed to moderate nonproliferative DR or worse DR in 23 subjects. On Cox proportional hazards regression analysis, HbA1c ARV, but not HbA1c CV, was significantly associated with DR development. However, the association between HbA1c variability and the DR progression rate to moderate nonproliferative DR or worse DR was not significant. The inter-visit HbA1c difference value on consecutive examination predicted DR development well and more careful screening for DR is needed for those with an absolute value change of 2.05%, an absolute increase of 1.75%, and an absolute decrease of 1.45% in HbA1c levels on consecutive examination. These results indicate that long-term glucose variability measured by HbA1c ARV might be an independent risk factor for DR development in addition to the mean HbA1c level in early diabetic subjects.

## Introduction

Diabetic retinopathy (DR) is the leading cause of blindness in adults living in developed countries. DR is a microvascular complication that occurs in about 35% of subjects with diabetes^1,2^. Chronic hyperglycemia and a long duration of diabetes are among the most important risk factors for the development and progression of DR; thus, lowering blood glucose levels in subjects with diabetes is crucial. Recently developed diabetic medications have aided in controlling subjects’ blood glucose levels; however, contrary to expectation, a few studies have recently reported higher mortality rates in an intensive-glucose control group^3^. Hypoglycemia and hyperglycemia following hypoglycemia are thought to be the main cause of the higher mortality rate in this group. Thus, the importance of the glycemic variability in diabetes complications is gaining attention, and the effects of glycemic variability on the association with diabetic nephropathy and cardiovascular events in type 1 diabetes subjects^4^ as well as in type 2 diabetes^5–7^ have been reported.

Several studies have reported that glycemic variability is associated with DR development and progression in type 1 diabetes subjects. Kilpatrick et al. analyzed data from the Diabetes Control and Complications Trial and showed that, by adding information about HbA1c variability on mean HbA1c, the predictability of DR development and progression improved^8^. A recent study by Hietala et al. confirmed that HbA1c variability was associated with an increased risk of retinopathy requiring laser treatment in subjects with type 1 diabetes^9^. Hermann et al. also reported that increased HbA1c variability was associated with a higher risk of DR^10^.

However, the relationship between glycemic variability and DR in type 2 diabetes subjects has not yet been clarified. Gimeno-Orna et al. found that fasting plasma glucose (FPG) variability was predictive of DR onset, irrespective of HbA1c levels^11^. Takao et al. revealed that the standard deviation (SD) of FPG is a risk factor for the development of DR, independent of the mean FPG or HbA1c level in type 2 diabetes subjects^12,13^. On the other hand, Zoppini et al. reported that FPG variability was not an independent risk factor for development and progression of DR^14^. Penno et al. revealed that HbA1c SD was related to the prevalence of diabetic nephropathy, but not to that of DR^15^. In a recent study of Asian subjects with type 2 diabetes, Foo et al. revealed that HbA1c variability was not associated with the presence of moderate DR^16^.

Most previous studies of subjects with type 2 diabetes used FPG variability as an indicator of glycemic variability, however, FPG has a limitation in that it does not reflect postprandial glucose levels, which has recently been considered important in terms of diabetes control. HbA1c can be a better indicator in that it reflects both FPG and postprandial glucose levels^17^. Besides, previous studies used SD or coefficient of variation (CV) values as an index for glucose variability. These variability indexes have a pitfall in that these only reflect the dispersion of the measurements around a single value (the mean) not considering the order of the measurements obtained^18^. The average real variability (ARV) is an indicator that reflects the sum of variability between two successive measurements and is known to be a more reliable representation of variability than SD^18^. Thus, in this retrospective cohort study, we investigated whether long-term HbA1c variability which was assessed by ARV and CV of HbA1c, is associated with the development of DR in type 2 diabetes subjects.

## Results

**Figure 1:**
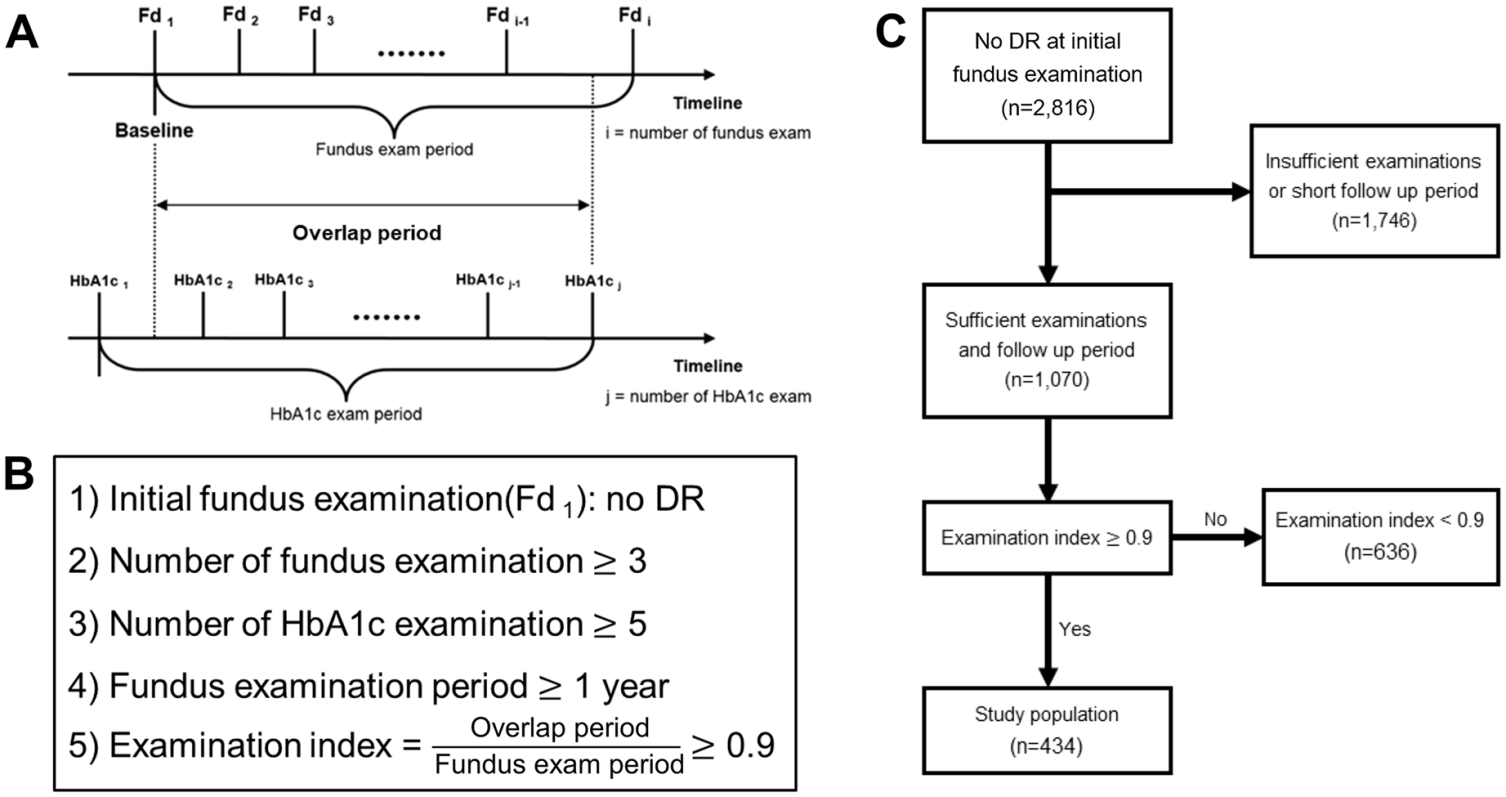
Schematic diagram for study design, inclusion criteria, and study flow chart. (**A**) Study design and timeline of the study. The date of the first fundus examination was designated as the baseline. The overlap period was defined as the length of the period when both serial fundus examination for diabetic retinopathy (DR) screening and serial HbA1c measurement were performed. (**B**) Inclusion criteria. The examination index was defined as the ratio of the overlap period to the fundus examination period, and in this study, only those with an examination index over 0.9 were included. (**C**) Study flow chart. After excluding those with insufficient examinations or a short follow-up period, 434 subjects were finally enrolled in this study.

A total of 434 subjects met the inclusion criteria and were finally enrolled in this study. The schematic diagram for study design and study flow chart are summarized in Fig. 1.

Eighty-six subjects were reported to have any degree of DR development during follow-up in the medical chart, however, we could confirm diabetic fundus changes such as microaneurysms, retinal hemorrhages, cotton wool spots, or hard exudates only in 55 subjects through the wide-field fundus photography which was taken on average, 2.9 ± 2.2 years later from the initial diagnosis. In this study, we defined these fundus photography-confirmed 55 subjects as DR development cases and excluded 31 subjects with possible misdiagnosis or transient fundus changes from DR development cases. The DR developed 3.4 ± 1.9 (range, 0.1–8.0) years from baseline fundus examination and 11.3 ± 4.6 (range, 1.6–26.6) years from diabetes onset. Twenty-three subjects showed progression to moderate nonproliferative DR (NPDR) or worse DR in one or both of their eyes during follow-up. DR progressed to moderate NPDR or worse DR 3.9 ± 2.3 (range, 0.8–9.4) years from baseline fundus examination and 11.9 ± 4.1 (range, 1.6–19.6) years from diabetes onset. There were no significant differences in age or sex between those with and without DR development. Those with DR development showed higher mean HbA1c (DR development 8.1 ± 1.0% vs. No DR development 7.3 ± 0.8%, *p* < 0.001) and higher HbA1c variability (ARV: DR development 0.78 ± 0.47% vs. No DR development 0.54 ± 0.29%, *p* < 0.001; CV: DR development 11.4 ± 5.9% vs. No DR development 9.5 ± 4.6%, *p* = 0.004) compared to no DR development group. Those with DR development tried and used more numerous types of anti-diabetic medication compared to no DR development subjects (DR development 3.8 ± 1.3 types vs. No DR development 3.1 ± 1.2 types, *p* < 0.001). In particular, the proportion of insulin (61.8% vs. 44.1%, *p* = 0.014), sulfonylurea (78.2% vs. 64.4%, *p* = 0.043), and sodium-glucose co-transporter-2 inhibitor (25.5% vs. 14.8%, *p* = 0.044) use was higher in DR development subjects. Those with DR development underwent fundus examination for a longer period compared to the no DR development group (DR development 6.6 ± 2.0 years (median 6.9 years) vs. no DR development 5.8 ± 1.8 years (median 5.8 years), *p* = 0.002, Table 1).

**Table 1:** Demographics and clinical characteristics of the subjects classified according to the development of diabetic retinopathy during follow-up.

	No DR development (N = 379)	DR development (N = 55)	*p*-value*
Age, years	57.5 ± 9.8	56.6 ± 11.1	0.512
Male, n (%)	205 (54.1)	33 (60.0)	0.410
Diabetes duration, years	6.7 ± 5.8	8.1 ± 5.0	0.095
Hypertension, n (%)	250 (66.0)	35 (63.6)	0.734
Type of anti-diabetic medications^†^	3.1 ± 1.2	3.8 ± 1.3	< 0.001
**Anti-diabetic medications, n (%)**			
Insulin	167 (44.1)	34 (61.8)	0.014
Biguanide	365 (96.3)	53 (96.4)	> 0.999
Thiazolidinedione	55 (14.5)	13 (23.6)	0.082
GLP-1 receptor agonist	7 (1.8)	3 (5.5)	0.121
Sulfonylurea	244 (64.4)	43 (78.2)	0.043
DPP-4 inhibitor	274 (72.3)	46 (83.6)	0.074
SGLT2 inhibitor	56 (14.8)	14 (25.5)	0.044
**Anti-HT medication, n (%)**			
Calcium channel blocker	164 (43.3)	25 (45.5)	0.760
ARB/ACEi	214 (56.5)	33 (60.0)	0.621
Beta blocker	131 (34.6)	13 (23.6)	0.108
Alpha blocker	4 (1.1)	0	> 0.999
Statin, n (%)	320 (84.4)	43 (78.2)	0.242
BMI, kg/m^2^	25.6 ± 3.4	25.3 ± 3.2	0.609
Hemoglobin, g/dL	13.7 ± 1.6	14.0 ± 1.6	0.198
Creatinine, mg/dL	0.87 ± 0.34	0.84 ± 0.19	0.490
LDL, mg/dL	99.5 ± 30.0	100.8 ± 27.1	0.763
HDL, mg/dL	49.1 ± 12.1	50.0 ± 14.3	0.624
Triglyceride, mg/dL	142.7 ± 83.0	167.9 ± 94.8	0.051
Mean HbA1c, %	7.3 ± 0.8	8.1 ± 1.0	< 0.001
HbA1c ARV, %	0.54 ± 0.29	0.78 ± 0.47	< 0.001
HbA1c CV, %	9.5 ± 4.6	11.4 ± 5.9	0.004
Number of fundus exam	7.2 ± 4.6	10.6 ± 6.0	< 0.001
Duration of fundus exam, years	5.8 ± 1.8	6.6 ± 2.0	0.002
Number of HbA1c exam	24.6 ± 8.0	26.8 ± 8.9	0.052
Duration of HbA1c exam, years	7.5 ± 1.4	7.9 ± 1.4	0.067

*Student’s *t*-test for continuous variables and chi-square test for categorical variables.

Abbreviations: Anti-HT, anti-hypertensive; ARB/ACEi, angiotensin receptor blocker/angiotensin-converting enzyme inhibitor; ARV, average real variability; BMI, body mass index; CV, coefficient of variation; DPP4, dipeptidyl peptidase-4; DR, diabetic retinopathy; GLP-1, glucagon-like peptide-1; HbA1c, hemoglobin A1c; HDL, high-density lipoprotein; LDL, low-density lipoprotein; SGLT2, sodium-glucose co-transporter-2.

^†^Number of different types of anti-diabetic medications including both oral hypoglycemic agents and insulin which has been used during the follow-up period.

**Figure 2:**
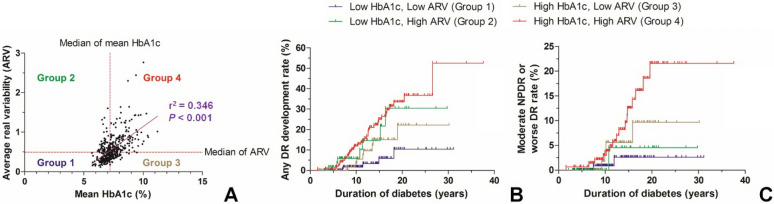
Comparison of development and progression rate of diabetic retinopathy (DR) according to the mean and average real variability (ARV) of HbA1c level. (**A**) Subject grouping according to their mean and ARV value of HbA1c. The mean and ARV of HbA1c showed a significant positive correlation (r = 0.588, *p* < 0.001). (**B**,**C**) Comparison of the rate of the DR development (**B**) and DR progression to moderate nonproliferative DR or worse DR (**C**) in four groups.

The mean HbA1c and HbA1c ARV were significantly and positively correlated on Pearson’s correlation test (r = 0.588, *p* < 0.001; Fig. 2A). We divided subjects into 4 groups according to their mean HbA1c and HbA1c ARV values. The demographic and clinical characteristics of these 4 groups are compared in Table 2. There were no significant differences in terms of age and sex between those with low mean HbA1c and low HbA1c ARV (Group 1) and those with low mean HbA1c and high HbA1c ARV (Group 2) and between those with high mean HbA1c and low HbA1c ARV (Group 3) and those with high mean HbA1c and high HbA1c ARV (Group 4). Those groups with higher HbA1c ARV showed a higher rate of insulin treatment compared to their low HbA1c ARV counterparts (Group 2, 47.8% vs. Group 1, 30.0%, *p* = 0.012; Group 4, 63.3% vs. Group 3, 43.3%, *p* = 0.006). Those groups with higher HbA1c ARV showed a higher rate of DR development with borderline significance (Group 2, 10.4% vs. Group 1, 3.3%, *p* = 0.051; Group 4, 23.3% vs. Group 3, 11.9%, *p* = 0.052). However, there were no significant differences in the proportion of progression to moderate NPDR or worse DR between those with high and low HbA1c ARV (Table 2).

**Table 2:** Demographics and clinical characteristics of the subjects classified according to the mean HbA1c and the HbA1c variability.

	Group 1 Low mean HbA1c, Low HbA1c ARV (N = 150)	Group 2 Low mean HbA1c, High HbA1c ARV (N = 67)	*p*-value* Group 1 vs Group 2	Group 3 High mean HbA1c, Low HbA1c ARV (N = 67)	Group 4 High mean HbA1c, High HbA1c ARV (N = 150)	*p*-value* Group 3 vs Group 4
Age, years	58.6 ± 10.0	58.6 ± 10.3	0.975	57.0 ± 7.6	55.8 ± 10.6	0.400
Male, n (%)	77 (51.3)	40 (59.7)	0.253	33 (49.3)	88 (58.7)	0.197
Diabetes duration, years	6.0 ± 5.4	4.7 ± 5.7	0.103	8.5 ± 5.1	8.1 ± 5.8	0.660
Hypertension, n (%)	105 (70.0)	49 (73.1)	0.638	40 (59.7)	91 (60.7)	0.893
**Anti-diabetic medications, n (%)**						
Insulin	45 (30.0)	32 (47.8)	0.012	29 (43.3)	95 (63.3)	0.006
Biguanide	147 (98.0)	65 (97.0)	0.645	67 (100)	139 (92.7)	0.020
Thiazolidinedione	13 (8.7)	5 (7.5)	0.766	10 (14.9)	40 (26.7)	0.058
GLP-1 receptor agonist	1 (0.7)	0	> 0.999	3 (4.5)	6 (4.0)	> 0.999
Sulfonylurea	76 (50.7)	43 (64.2)	0.065	52 (77.6)	116 (77.3)	0.964
DPP-4 inhibitor	95 (63.3)	52 (77.6)	0.038	50 (74.6)	123 (82.0)	0.212
SGLT2 inhibitor	15 (10.0)	9 (13.4)	0.456	12 (17.9)	34 (22.7)	0.428
**Anti-HT medication, n (%)**						
Calcium channel blocker	66 (44.0)	36 (53.7)	0.185	24 (35.8)	63 (42.0)	0.391
ARB/ACEi	94 (62.7)	39 (58.2)	0.533	35 (52.2)	79 (52.7)	0.954
Beta blocker	50 (33.3)	25 (37.3)	0.569	18 (26.9)	51 (34.0)	0.297
Alpha blocker	2 (1.3)	0	> 0.999	1 (1.5)	1 (0.7)	0.523
Statin, n (%)	132 (88.0)	52 (77.6)	0.049	57 (85.1)	122 (81.3)	0.503
BMI, kg/m^2^	26.1 ± 3.3	25.4 ± 3.7	0.281	26.7 ± 3.3	24.8 ± 3.1	0.003
Hemoglobin, g/dL	13.7 ± 1.5	13.7 ± 1.9	0.900	13.8 ± 1.7	13.8 ± 1.5	0.785
Creatinine, mg/dL	0.87 ± 0.42	0.86 ± 0.33	0.947	0.85 ± 0.22	0.88 ± 0.25	0.357
LDL, mg/dL	100.6 ± 28.9	91.3 ± 33.4	0.061	102.6 ± 26.9	100.7 ± 29.7	0.682
HDL, mg/dL	49.5 ± 11.2	46.9 ± 13.9	0.224	51.6 ± 12.5	48.8 ± 12.9	0.182
Triglyceride, mg/dL	145.8 ± 78.9	127.2 ± 68.7	0.134	151.8 ± 110.0	151.8 ± 84.0	0.999
Mean HbA1c, %	6.7 ± 0.4	6.8 ± 0.3	0.091	7.6 ± 0.4	8.2 ± 0.8	< 0.001
HbA1c ARV, %	0.32 ± 0.08	0.68 ± 0.22	< 0.001	0.40 ± 0.07	0.84 ± 0.36	< 0.001
HbA1c CV, %	6.5 ± 2.2	12.9 ± 5.5	< 0.001	7.4 ± 2.3	12.6 ± 4.7	< 0.001
Number of fundus exam	7.3 ± 4.5	6.9 ± 4.8	0.553	8.1 ± 4.7	8.1 ± 5.4	0.987
Duration of fundus exam, years	5.8 ± 1.6	5.1 ± 1.9	0.006	6.1 ± 1.4	6.3 ± 2.1	0.560
Number of HbA1c exam	24.0 ± 7.8	19.8 ± 7.8	< 0.001	27.7 ± 5.5	26.7 ± 8.5	0.298
Duration of HbA1c exam, years	7.4 ± 1.3	6.9 ± 1.8	0.035	7.8 ± 0.8	7.9 ± 1.4	0.598
Any DR development, n (%)	5 (3.3)	7 (10.4)	0.051	8 (11.9)	35 (23.3)	0.052
≥ Mod NPDR progression, n (%)	2 (1.3)	1 (1.5)	> 0.999	4 (6.0)	16 (10.7)	0.269

* Student’s *t*-test for continuous variables and chi-square or Fisher’s exact test for categorical variables.

Abbreviations: Anti-HT, anti-hypertensive; ARB/ACEi, angiotensin receptor blocker/angiotensin-converting enzyme inhibitor; ARV, average real variability; BMI, body mass index; CV, coefficient of variation; DPP4, dipeptidyl peptidase-4; DR, diabetic retinopathy; GLP-1, glucagon-like peptide-1; HbA1c, hemoglobin A1c; HDL, high-density lipoprotein; LDL, low-density lipoprotein; Mod NPDR, moderate nonproliferative diabetic retinopathy; SGLT2, sodium-glucose co-transporter-2.

The overall DR development and DR progression rate (progression to moderate NPDR or worse DR) were significantly different among these groups. Group 4 and Group 2 showed a rapid rate of DR development compared to Group 1 (Group 4 vs. Group 1, *p* < 0.001; Group 2 vs. Group 1, *p* = 0.008, Log-rank test, Fig. 2B). On the comparison of DR progression rate, Group 4 showed rapid progression compared to Group 1 (*p* = 0.010, Log-rank test, Fig. 2C).

On Cox proportional hazards regression analysis, age (HR 0.973, *p* = 0.044), diabetes duration (HR 0.807, *p* < 0.001), insulin (HR 1.692, *p* = 0.059), sodium-glucose co-transporter-2 inhibitor (HR 1.752, *p* = 0.072), hemoglobin (HR 1.242, *p* = 0.020), triglyceride (HR 1.004, *p* = 0.006), mean HbA1c (HR 1.898, *p* < 0.001), HbA1c ARV (HR 6.206, *p* < 0.001), HbA1c CV (HR 1.130, *p* < 0.001) were associated with DR development with *p* < 0.1. These factors were entered into multivariate analysis (HbA1c ARV and HbA1c CV were entered separately as an index of HbA1c variability). Only diabetes duration (HR 0.789, *p* < 0.001), mean HbA1c (HR 1.672, *p* = 0.008), and HbA1c ARV (HR 2.479, *p* = 0.036) showed statistically significant association with DR development on multivariate analysis. For analysis on DR progression to moderate NPDR or worse DR, only diabetes duration (HR 0.766, *p* = 0.001), insulin use (HR 3.646, *p* = 0.027), mean HbA1c (HR 1.819, *p* = 0.035) were significantly associated with DR progression whereas HbA1c ARV did not show significant association on multivariate analysis (Table 3).

**Table 3:** Cox proportional hazards regression analysis for factors associated with development and progression of diabetic retinopathy.

	Any DR development				Moderate NPDR or worse DR			
	HR (95% CI)	p-value	Adjusted HR* (95% CI)	p-value	HR (95% CI)	p-value	Adjusted HR* (95% CI)	p-value
Age, years	0.973 (0.947–0.999)	0.044	1.026 (0.992–1.061)	0.133	0.938 (0.904–0.974)	0.001	0.996 (0.952–1.042)	0.858
Male	1.337 (0.779–2.294)	0.292			1.178 (0.516–2.687)	0.698		
Diabetes duration, years	0.807 (0.742–0.877)	< 0.001	0.789 (0.715–0.870)	< 0.001	0.790 (0.690–0.903)	0.001	0.766 (0.659–0.890)	0.001
Hypertension	0.894 (0.516–1.551)	0.691			0.660 (0.289–1.510)	0.325		
Insulin	1.692 (0.981–2.920)	0.059	1.472 (0.774–2.802)	0.239	4.815 (1.636–14.169)	0.004	3.646 (1.155–11.508)	0.027
Thiazolidinedione	1.339 (0.718–2.496)	0.358			2.252 (0.954–5.318)	0.064	1.402 (0.524–3.750)	0.500
SGLT2 inhibitor	1.752 (0.952–3.224)	0.072	1.349 (0.663–2.746)	0.408	2.200 (0.903–5.359)	0.083	1.355 (0.460–3.995)	0.582
Statin	0.706 (0.372–1.340)	0.287			0.734 (0.272–1.980)	0.541		
BMI, kg/m^2^	0.998 (0.909–1.097)	0.974			1.035 (0.888–1.207)	0.660		
Hemoglobin, g/dL	1.242 (1.036–1.490)	0.020	1.079 (0.876–1.328)	0.476	1.245 (0.936–1.657)	0.132		
Creatinine, mg/dL	0.503 (0.129–1.955)	0.321			0.554 (0.075–4.093)	0.562		
LDL, mg/dL	1.002 (0.992–1.012)	0.703			1.001 (0.986–1.017)	0.868		
HDL, mg/dL	0.999 (0.976–1.022)	0.900			1.008 (0.975–1.043)	0.627		
Triglyceride, mg/dL	1.004 (1.001–1.006)	0.006	1.003 (1.000–1.006)	0.060	1.003 (0.999–1.007)	0.165		
Mean HbA1c, %	1.898 (1.471–2.448)	< 0.001	1.672 (1.146–2.439)	0.008	2.507 (1.707–3.683)	< 0.001	1.819 (1.044–3.171)	0.035
HbA1c ARV, %	6.206 (3.562–10.812)	< 0.001	2.479 (1.063–5.782)	0.036	6.656 (2.641–16.772)	< 0.001	1.596 (0.402–6.333)	0.506
HbA1c CV, %	1.130 (1.069–1.193)	< 0.001	1.036^†^ (0.966–1.111)	0.319	1.101 (1.008–1.204)	0.034	0.985^†^ (0.883–1.099)	0.787

*Adjusted hazard ratio (HR) was calculated from multivariate analysis using variables with p < 0.100 in univariate analysis. For those HbA1c variability indexes (HbA1c ARV and HbA1c CV), only HbA1c ARV was entered into the equation.

^†^Adjusted hazard ratio for HbA1c CV was calculated separately from that for HbA1c ARV, i.e., HbA1c CV value was entered into the multivariate equation instead of HbA1c ARV value.

ARV, average real variability; BMI, body mass index; CV, coefficient of variation; DR, diabetic retinopathy; HbA1c, hemoglobin A1c; HDL, high-density lipoprotein; HR, hazard ratio; LDL, low-density lipoprotein; NPDR, nonproliferative diabetic retinopathy; SGLT2, sodium-glucose co-transporter-2.

**Figure 3:**
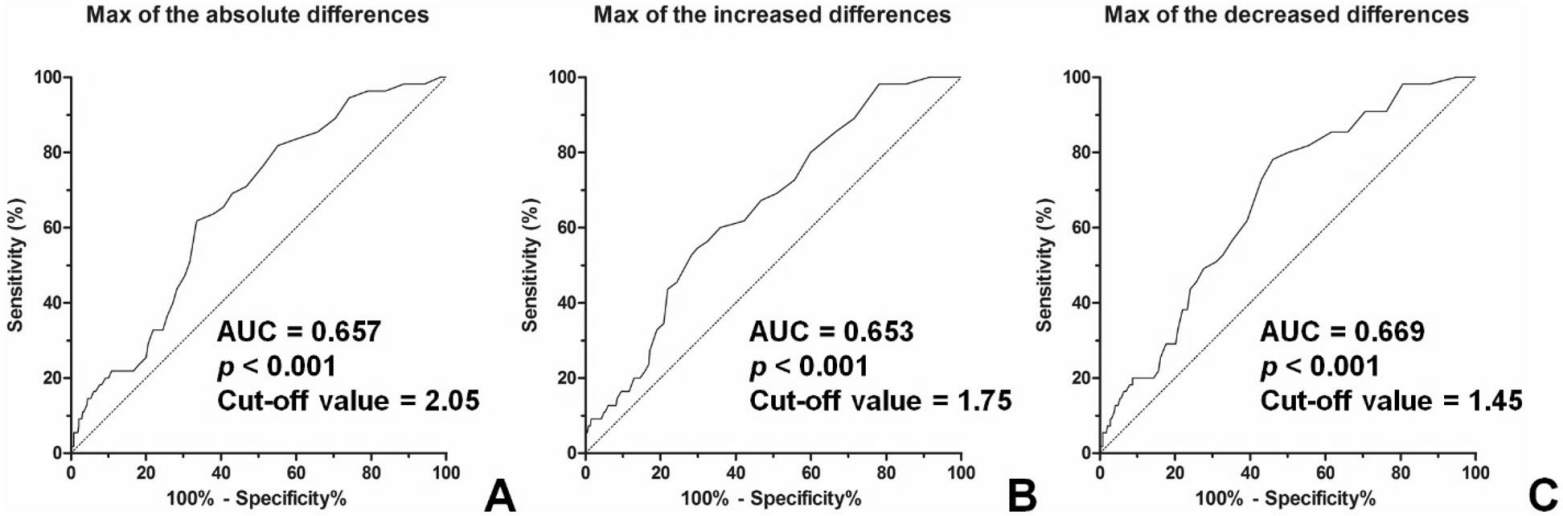
Receiver operating characteristic (ROC) curve for predicting diabetic retinopathy development using inter-visit HbA1c differences. (**A**) The ROC curve of the absolute inter-visit HbA1c level difference, (**B**) The ROC curve of the increase in HbA1c difference, (**C**) The ROC curve of the decrease in HbA1c difference. AUC, area under the curve.

We evaluated whether the inter-visit HbA1c difference on consecutive examination could predict DR progression using an analysis of the area under the receiver operating characteristic curve (AUC). The maximum value of absolute inter-visit HbA1c differences (AUC 0.657, 95% CI: 0.587–0.727, *p* < 0.001), the maximum value of the increased HbA1c difference (AUC 0.653, 95% CI: 0.582–0.725, *p* < 0.001), and the maximum value of the decreased HbA1c difference (AUC 0.669, 95% CI: 0.600–0.737, *p* < 0.001) in each subject were good predictors of DR development. The maximum sum of sensitivity and specificity was observed with an inter-visit HbA1c difference cut-off point of 2.05%, 1.75%, and 1.45% for absolute HbA1c difference, increased HbA1c difference and decreased HbA1c difference, respectively (Fig. 3).

## Discussion

In this study, we evaluated the influence of long-term glycemic variability on DR development and progression in type 2 diabetes subjects. The greater HbA1c variability which was measured using HbA1c ARV was an independent risk factor for new-onset DR development. Not only the increase in HbA1c level but also the abrupt decrease in HbA1c level was associated with DR development. However, the association between HbA1c variability and the DR progression rate to moderate NPDR or worse DR was not significant.

There are controversies on the relationship between glycemic variability and DR development or progression in type 2 diabetes subjects. Some researchers reported that higher glucose variability was associated with the risk of DR development or progression^11–13^. On the other hand, other researchers also suggest that glucose variability is not an independent risk factor for DR development or progression^14–16^. Our findings are interesting and lie between the above two arguments. The glucose variability measured by HbA1c ARV but not HbA1c CV showed a significant association with the new-onset DR development. On the other hand, the glucose variability was not associated with the DR progression to moderate NPDR or worse DR. These can be explained by the following reasons.

The HbA1c variability showed a significant and positive correlation with the mean HbA1c value. In those with a large mean HbA1c value, the variability is also large. The CV is calculated by dividing the SD by the mean value to correct this, however, there is still a significant positive correlation between the mean HbA1c and HbA1c CV. In our 4 group analysis, there was a significant difference in mean HbA1c between two high mean HbA1c groups; group 4 (high HbA1c ARV, high mean HbA1c) showed a significantly higher mean HbA1c level than group 3 (low HbA1c ARV, high mean HbA1c). The group with a large degree of HbA1c variability also showed a higher mean HbA1c, thus, there was a limitation in comparing the two groups in terms of glucose variability thoroughly. On the other hand, in those with low mean HbA1c levels, the correlation between mean HbA1c and HbA1c variability was relatively low, and it is thought that there is room for evaluating the influence of glucose variability on DR development independently. Group 2 (high HbA1c ARV, low mean HbA1c) showed a higher rate of DR development than group 1 (low HbA1c ARV, low mean HbA1c). Thus, when the blood glucose level is relatively low, glucose variability might act as an independent and additional risk factor for DR development. On the contrary, when the blood glucose level is high, the glucose variability also increases, and it is difficult to explore the independent influence of glucose variability on DR development.

Several reports have addressed the pathophysiology underlying the effects of glycemic variability on DR. First, in addition to persistent chronic hyperglycemia, transient high glucose spikes can cause epigenetic changes due to higher oxidative stress^19^. Second, glucose fluctuation damages endothelial function in microvascular and macrovascular beds^20^. Thus, oscillating glucose levels could induce endothelial dysfunction and change vessel wall morphology more than a consistently high glucose concentration could in itself^21,22^.

Interestingly, the glucose variability measured by HbA1c ARV was more sensitive to represent DR risk than that by HbA1c CV. ARV is considered to reflect the actual fluctuations more by considering the order of measurement. It has been widely studied in the field of cardiovascular diseases for the measurement of blood pressure variability and is considered to be a useful method for studying the clinical implication of blood pressure variability^18,23,24^. In the previous studies, glucose variability was measured with FPG ARV to evaluate its effect on cardiovascular disease and mortality^25^. Recently, Zhou et al. evaluated the association of glycemic variability which was measured by both FPG CV and FPG ARV with microvascular complications in type 2 diabetes subjects and found that both FPG CV and FPG ARV were associated with future microvascular outcomes^26^. However, studies on HbA1c ARV is lacking. HbA1c variability represents long-term glucose variability and further studies using this index on the association between long-term glucose variability and microvascular outcomes are needed.

In this study, a shorter duration of diabetes at baseline was associated with DR development and progression. This is the opposite of the general idea that the duration of diabetes is related to the prevalence and risk of DR^27^. We believe that this is due to our study design. We did not recruit subjects with newly diagnosed diabetes, but instead, we recruited those who underwent eye examinations in our retina clinic. Subjects had on average 6.9 years of diabetes on baseline eye examination, and those who showed DR on baseline examination were excluded from the study. Thus, the duration of diabetes in this study could be interpreted as the duration of the retinopathy-free period, and there is a possibility that those with a longer retinopathy-free period might have had better glucose control.

We also investigated information on various medications taken by the subjects. More numerous types of anti-diabetic drugs were used in subjects with DR development, and in particular, insulin use was significantly associated with progression to moderate NPDR or worse DR. This was not a prospective study, thus, there were no strict guidelines for drug use. However, in general, the more uncontrolled diabetes, the more types of drugs are tried and used^28,29^. Insulin is effective in glycemic control and it possesses a better ability in the preservation of β-cell function, however, it has a risk of hypoglycemia^30^. It is reported that insulin use was associated with an increased risk of all-cause mortality compared to the use of dipeptidyl peptidase-4 inhibitors or thiazolidinediones^31,32^. This study was not originally designed to evaluate the influence of medications on DR development and detailed information such as dosage or duration of drug use was not included. Further studies on the association between anti-diabetic medication and DR development are needed.

To measure the glucose variability, we need to calculate SD, CV, or ARV values of glucose levels, which is not implemented clinically. Instead, clinicians usually compare glucose levels with the previous measurements. In this study, we evaluated the inter-visit HbA1c level changes to see if the degree of increase or decrease in the level of consecutive HbA1c measurement could predict DR development. Interestingly, not only the increase in HbA1c but also the sudden decrease in HbA1c was associated with DR development. The suggested cut-off value for the abnormal HbA1c changes are 2.05%, 1.75%, and 1.45% for absolute HbA1c difference, increased HbA1c difference, and decreased HbA1c difference, respectively. Thus, physicians should pay attention to sudden decreases as well as increases in HbA1c levels of subjects when considering their DR risk.

Our study had some limitations. First, due to its retrospective design, we were not able to control the varying number of measurements per subject and various time intervals between examinations. Second, because DR grading was performed retrospectively through medical chart and fundus photography review, the grading could be inaccurate. Third, there were not many cases of DR development and progression, due to the relatively small number of cases and the short follow-up period. Fourth, subjects that are not adherent to follow-up visits were excluded from the study, and this could have excluded subjects at higher risk of poor glycemic control and complications from the study. Fifth, factors other than HbA1c levels, such as serial changes in blood pressure are lacking and we could not observe their variation throughout the follow-up.

In conclusion, long-term glycemic variability as measured by HbA1c ARV was associated with the development of new-onset DR in subjects with type 2 diabetes, independently of the mean value of HbA1c. However, the association between HbA1c variability and the DR progression rate to moderate NPDR or worse DR was not significant. HbA1c ARV might be an independent risk factor for DR development in addition to the mean HbA1c level in early diabetic subjects. More careful screening for DR is needed for those with an absolute value change of 2.05%, an increase of 1.75%, and a decrease of 1.45% in HbA1c levels on consecutive examination.

## Methods

This study was reviewed and approved by the institutional review board (IRB) of Kangdong Sacred Heart Hospital (IRB No. 2018-11-009). This study adhered to the tenets of the Declaration of Helsinki. Informed consent was waived by the Kangdong Sacred Heart Hospital IRB due to the retrospective nature of the study.

### Subjects

We retrospectively reviewed medical records of subjects with type 2 diabetes who underwent DR screening at Kangdong Sacred Heart Hospital Ophthalmology clinic from January 1, 2009, to July 31, 2017. The subjects’ first fundus examination date was set as the baseline. The inclusion criteria for subjects were as follows: 1) those without any clinical sign of DR in both eyes on initial examination, 2) those who underwent regular fundus examination for more than 1 year, 3) those who underwent at least 3 fundus examinations, 4) those who underwent at least 5 HbA1c level measurements, 5) regular HbA1c level measurement that covered at least 90% of the fundus examination follow-up period (Fig. 1). Exclusion criteria were as follows: 1) those with chorioretinal pathologies, such as age-related macular degeneration, retinal vein occlusion, retinal artery occlusion, hypertensive retinopathy, uveitis, etc., 2) those with corneal opacity or dense cataract prohibiting accurate retinal evaluation, 3) those who had undergone intraocular surgery except for uncomplicated cataract surgery.

### Ocular examination and data collection

We retrospectively reviewed the medical records of the subjects for their fundus findings and laboratory examination results. We collected every HbA1c measurement during the follow-up period. HbA1c was assessed by ion-exchange high-performance liquid chromatography (D-100; Bio-Rad, Los Angeles, CA, USA), using an assay that was accredited by the National Glycoprotein Standardization Program. In addition to HbA1c, hemoglobin, low-density lipoprotein (LDL) cholesterol, high-density lipoprotein (HDL) cholesterol, triglyceride, and creatinine levels at the baseline were collected. We also collected body mass index (BMI), diabetes duration, and information on medications that were prescribed during the follow-up period. Hypertensive cases were defined as those who were diagnosed to have hypertension at the medical clinic and taking anti-hypertensive drugs. The average HbA1c level of the whole follow-up period was calculated (mean HbA1c). We used the CV and ARV of HbA1c as the indicator of the long-term glycemic variability: For CV calculation, intra-individual standard deviation (SD) was divided by mean HbA1c to correct for higher SDs due to larger absolute values. CV was further divided by the square root of the ratio of HbA1c measurements (N) to N-1, $\sqrt{N/(N-1)}$, to account for the possible influence on the CV of the HbA1c measurement number^8,25^. The ARV is defined as^18^,

$$ARV= \frac{1}{N-1}\sum_{k=1}^{N-1}\left|{HbA1c}_{k+1}-{HbA1c}_{k}\right|$$

Before April 2017, fundus photographs were obtained using a 45° digital retinal camera (Topcon TRC-NW8; Topcon, Oakland, NJ, USA) and after 2017, fundus photographs were obtained using an ultra-widefield retinal imaging device (Optos 200Tx; Optos plc, Dunfermline, UK), for each subject at each visit. Subjects underwent the fundus examination using an indirect ophthalmoscope. DR was graded at each visit as one of the following gradings; no DR, mild NPDR, moderate NPDR, severe NPDR, and proliferative DR according to the international clinical diabetic retinopathy disease severity scale^33^. In this study, we considered DR progressed cases as those who were reported to have DR in the medical chart and which was also confirmed in the widefield fundus photograph which was taken on average, 2.9 ± 2.2 years later from the first diagnosis. Thus, some of the subjects with transient early diabetic changes such as microaneurysms and dot hemorrhages that have disappeared on follow-up examinations were not considered as DR cases. The fundus photograph was reviewed by two examiners (H.U.K. and Y-K.K.) independently and when there was a discrepancy on DR grading between the two examiners, images were reviewed and reassessed together by both examiners, and an agreement was reached.

### Statistical analysis

We measured the correlation between mean HbA1c and HbA1c ARV using Pearson’s correlation test. We divided subjects according to their mean HbA1c and HbA1c ARV values. Subjects were divided into four groups based on cut-off values of the median value of mean-HbA1c and the median value of the HbA1c ARV. Log-rank test was performed to compare the DR development and progression rate among different groups. We also performed Cox proportional hazards regression analysis to investigate factors associated with DR development and progression. Clinical factors with a *p*-value < 0.1 on univariate analysis were included in multivariate analysis. Age, diabetes duration, insulin use, sodium-glucose co-transporter-2 use, hemoglobin, triglyceride, mean HbA1c, and HbA1c variability (either HbA1c ARV or HbA1c CV) were used for new-onset DR development evaluation and age, diabetes duration, insulin use, thiazolidinedione use, sodium-glucose co-transporter-2 use, mean HbA1c, and HbA1c variability (either HbA1c ARV or HbA1c CV) were entered into the equation for DR progression to moderate NPDR or worse DR evaluation. The absolute value of the inter-visit HbA1c differences for every visit was collected for each subject and the maximum value of the absolute inter-visit difference in HbA1c levels, the maximum value of the increased HbA1c difference and the maximum value of the decreased HbA1c difference were calculated for each subject. We performed receiver operating characteristic curve analysis using these inter-visit HbA1c difference values for predicting DR development. Statistical significance was defined as a *p*-value < 0.05, and borderline significance was defined as a *p*-value < 0.07.

## Data Availability

The datasets generated during the current study are available from the corresponding author upon request.
